# Modeling and Assessment of Ammonia Direct Reduction for Decarbonizing Iron Production

**DOI:** 10.1021/acsomega.6c03735

**Published:** 2026-07-10

**Authors:** Xuesong Lu, Dorcas Tuitoek, Binjian Nie, Aidong Yang

**Affiliations:** Department of Engineering Science, 6396 University of Oxford, Parks Road, Oxford OX1 3PJ, U.K.

## Abstract

Ammonia’s favorable properties for transport and storage make it a promising alternative to hydrogen as a low-carbon reductant for green steel, particularly in regions lacking renewable energy. Despite several experimental investigations, the systematic evaluation of iron ore reduction by ammonia in the shaft furnace is still challenging, as there is no comprehensive mathematical model to simulate its chemical and physical performance. In this study, a one-dimensional plug-flow model was first developed to describe the ammonia reduction process in an industrial-scale shaft furnace with counter-current gas and solid flows. The kinetics of chemical conversions, including ammonia decomposition, direct reduction by ammonia and hydrogen, and iron nitridation, were incorporated into the model, along with mass and heat transfer, within the framework of a grain model at the pellet scale. Calibrated by TGA experimental data, the effects of process conditions and gas recycling were systematically investigated through numerical simulation. The results indicate that the process of ammonia-based reduction of iron ore is strongly endothermic, making intensive heat supply essential. A high inlet gas temperature above 900 °C is favorable to the operation of the shaft furnace with a high metallization rate and a low nitridation rate. By introducing a purge stream, excess nitrogen and water vapor can be removed from the system, enabling gas recycling and the reuse of ammonia and hydrogen from the off-gases; this achieves a metallization rate exceeding 0.90 with a nitridation rate below 0.10. By comparing it with hydrogen-based reduction of iron ore, this work provides a deep understanding of ammonia reduction and offers valuable guidance for industrial-scale reactor design and supply chain analysis.

## Graphical abstract

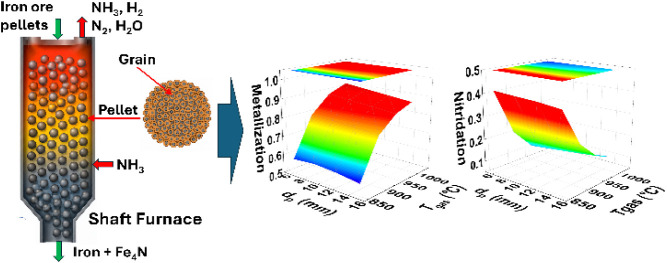

## 1 Introduction

CO_2_ emissions from industrial activities are among the major contributors to climate change. Current carbon emissions from the iron and steel industry account for 7–9% of the global carbon emissions, reaching up to 2.6 Gt per year.^1^ To resolve this problem, new advanced technological processes are increasingly being explored to achieve the target of zero-carbon emissions, contributing to the world’s carbon reduction plans. One such option is the use of hydrogen in iron ore reduction to replace carbon-intensive reductants.^2^ This option however has several challenges, such as high cost of green hydrogen generation, costly hydrogen transportation and storage, and higher safety requirements.

To address these challenges, iron ore reduction by ammonia (referred to as ammonia direct reduction, or ADR) has received increasing attention as an alternative to hydrogen direct reduction (HDR).^3^ With hydrogen constituting 17.6 wt %, ammonia is a desirable hydrogen carrier. Liquid ammonia has a 1.5–1.7 times higher volumetric density of hydrogen (120 kgH_2_/m^3^) than the liquid hydrogen (70 kgH_2_/m^3^), and it can be liquefied at −33.6 °C under 1 bar, or at 10 bar at ambient temperature, while hydrogen must be liquefied at −252.9 °C. These advantages make ammonia more suitable for storage and transportation. The comparison between hydrogen and ammonia is summarized in Table 1.^3–7^

**Table 1:** Comparison between Hydrogen and Ammonia

Catalogue	Hydrogen	Ammonia
Production cost (US dollar/kg)	3.5–6.0	0.48–1.22
Gravimetric energy density (kWh/kg)	33.3	5.3
Theoretical energy consumption for reducing iron ore (hematite) (MJ/kg (Fe)) based on reaction heat from reaction formula	0.88	1.71
(3/2) H_2_ + (1/2) Fe_2_O_3_ = Fe +(3/2) H_2_O		
NH_3_ + (1/2) Fe_2_O_3_ = Fe + (3/2) H_2_O + (1/2) N_2_		
Pipeline transportation cost (US dollar/km/ton)	0.57	0.11
Storage cost (US dollar/kg) (15 days)	1.97	0.06
Storage cost (US dollar/kg) (182 days)	14.95	0.54
Volumetric energy density (GJ/m^3^)	4.7	11.3
Flammability limit (%)	4.7–75	15–28
Autoignition temperature (°C)	560	651
Liquified temperature at atmospheric pressure (°C)	–252.9	–33.6
Stoichiometric coefficient of reducing gas for producing 1 mol Fe	1.5	1

Similar to reduction by hydrogen, the process of ADR for the most common type of iron ore, i.e., hematite (Fe_2_O_3_), occurs in three successive stages: hematite to magnetite (Fe_3_O_4_), magnetite to wustite (FeO), and finally wustite to metallic iron (Fe). However, ADR involves a unique aspect, namely iron nitridation. Iron can react with ammonia to form iron nitrides at a certain temperature, including particularly Fe_4_N, Fe_3_N, and Fe_2_N.^8^ The presence of nitrides can be reduced at a temperature higher than 650 °C, where the iron nitrides will start to decompose, through which metallic iron is recovered. Besides, catalyzed by the metallic iron as the product of ADR, ammonia can be decomposed to produce hydrogen, which then contributes to the reduction process. The research on ADR started in 2010s. Hosokai et al.^9^ investigated the reduction of hematite powder by ammonia for ironmaking under the effect of temperature in a fixed-bed reactor. The results showed that the reduction process began at 430 °C, a much lower temperature than in conventional carbon-based ironmaking. Yasuda et al.^10^ further investigated the reduction by ammonia in a fixed bed using hematite powders. They found that the ammonia started to decompose at 600 °C, triggered by the generation of α-Fe. In the temperature range from 520 to 590 °C, hematite was directly reduced to magnetite by ammonia, and then magnetite was reduced to iron by hydrogen generated from the decomposition of ammonia. The formation of nitrides was affected not only by the temperature but also by the nitridation potential, which is defined as *p*_NH3_/*p*_H2_^1.5^.

More recently, ADR has received significant attention. Iwamoto et al.^11^ investigated the effects of different ore types (i.e., high- and low-combined water ores), reduction temperatures (i.e., 650, 700, and 750 °C), and conditions of postreduction treatments (i.e., quenching by NH_3_, fast- and slow- quenching by inert gas) on ore reduction behaviors in a fixed-bed reactor. They discovered that the quenching conditions significantly affect the generated phases (Fe, FeO_*x*_, FeN_*y*_); these phases could be easily controlled in the postreduction process. Ma et al.^12^ performed reduction experiments using commercial hematite pellets in a thermogravimetric analysis (TGA) furnace and indicated that during the reaction, the generated porous iron can catalyze the decomposition of ammonia at elevated temperatures to release hydrogen for the reduction of iron oxides. Ammonia-based reduction of iron oxide was found to proceed through an autocatalytic reaction, kinetically as effective as hydrogen-based direct reduction, yielding the same metallization rate. They contemplated that the produced iron/iron nitride mixture could be subsequently melted in an electric arc furnace (or cocharged into a converter) to adjust the chemical composition to the target steel grades. Jovičević-Klug et al.^13^ investigated the reduction kinetics and nitridation of iron oxides exposed to ammonia at temperatures from 500 to 800 °C using pellets in a TGA system. It was shown that at temperatures below 600 °C, in situ nitridation accompanied the reduction process (which was also confirmed by a later study by Luu et al.^14^), forming predominantly Fe_3_N-type nitrides; at higher temperatures above 600 °C, ammonia decomposition became more prominent, leading to fast kinetics of iron ore reduction (comparable to HDR) and retardation of in situ nitridation due to the thermal instability of the nitrides. Mittal and Davis^15^ showed that increasing the reaction temperature to 900–1000 °C induced faster reaction rates, making reduction complete in 1 min with Fe_2_O_3_ powder on a quartz boat in a tube furnace. These higher temperatures improved the consumption ratio of the ammonia supplied up to 56%. The positive impact of operating at a higher temperature, along with a higher concentration of ammonia, was also confirmed by Liu et al.^16^ Furthermore, Liu et al.^17–19^ and Li et al.^20^ published several studies on ADR, covering gas adsorption, reaction characteristics, reduction kinetics, and iron nitridation.

On reaction kinetics of ammonia reduction of iron ores, Mukherjee et al.^21^ investigated ammonia direct reduction at the bench scale; the obtained activation energy for ammonia-based reduction was 41.76 kJ/mol. Liu et al.^17^ estimated the apparent activation energy for ammonia reduction to be 76.7 kJ/mol. Li et al.^22^ reported the apparent activation energy for the mixed gases of NH_3_ and CO ranging from 23.92 to 60.51 kJ/mol, which is lower than values obtained for pure NH_3_ or CO systems. However, the detailed reaction kinetics for individual reduction reactions from hematite to iron by ammonia have not been investigated extensively.

The experimental results of ADR reported so far have provided important empirical evidence regarding the key steps of the reduction process, such as ammonia decomposition, direct reduction by ammonia, reduction by hydrogen derived from ammonia, and nitridation. However, further systematic analyses of the behavior and performance of the ADR process at an industrially relevant scale require a numerical simulation model, which to the best of our knowledge has not been reported. Different from conventional ironmaking that uses blast furnaces, direct reduction of iron ore has been suggested to take place in a shaft furnace (moving bed) or a fluidized bed.^23^ Building on existing models of shaft furnace-based HDR,^24^ particularly the grain model^24–26^ that extends the traditional shrinking core model,^27,28^ the current work presents a shaft furnace model for ADR and demonstrates its usage in studying important process details, such as temperature, pressure, feeding rate of iron ore, and reducing gas composition, and in providing a coupled understanding of reaction kinetics, mass transfer, and heat transfer through numerical simulation. The key scale-up difficulties of the shaft furnace lie in the strong interactions between complex fluid dynamics, mass transfer, heat transfer, and reaction kinetics. Therefore, the modeling of iron ore reduction by ammonia in the shaft furnace is intended to improve the understanding of the essential physical and chemical behaviors, which in turn will support the development of up-scaled processes and equipment. The novelty of this paper lies in the development of the first comprehensive shaft furnace model for the reduction of iron ore by using ammonia. It thus provides a theoretical modeling basis for future assessment and development of ADR technology.

## 2 Theory

### 2.1 Model Overview and Assumptions

The ADR process taking place in a shaft furnace is schematically illustrated in Figure 1. The furnace comprises the iron ore feeding section, reduction section (the focus of this work) and cooling section. The solid iron ore pellets, assumed to be hematite (Fe_2_O_3_), are fed at an ambient temperature (∼30 °C) into the furnace from the top. The reducing gas, preheated to a high temperature (∼950 °C)^29–31^ is introduced into the furnace from a lower section of the reactor, thus forming counter-current two-phase flows in the furnace. Ammonia decomposition without catalysts can occur at the chosen feed temperature,^32^ although it is expected to have limited conversion^33^ and a low reaction rate.^34^ Thus, it can be seen that ammonia may undergo partial decomposition during the heating process when the inlet gas temperature increases to 950 °C. This decomposition could have some influence on the shaft furnace operation. However, in the present study, this effect is neglected because ammonia cracking is relatively slow in the absence of a catalyst, and the primary focus of this work is the reaction kinetics within the shaft furnace rather than the gas-heating process. It should be noted that the presence of N_2_ and H_2_ along with NH_3_ in the shaft furnace feed and its impact on the reduction process are considered in the gas-recycling case (Section 4.4). Therefore, ammonia cracking prior to the shaft furnace is not considered in this work. The operation is performed in the regime of a moving bed, with the gas velocity maintained below the minimum fluidization velocity. In the furnace, hematite gradually reduces to magnetite (Fe_3_O_4_), wustite (FeO), and iron (Fe).

**Figure 1:**
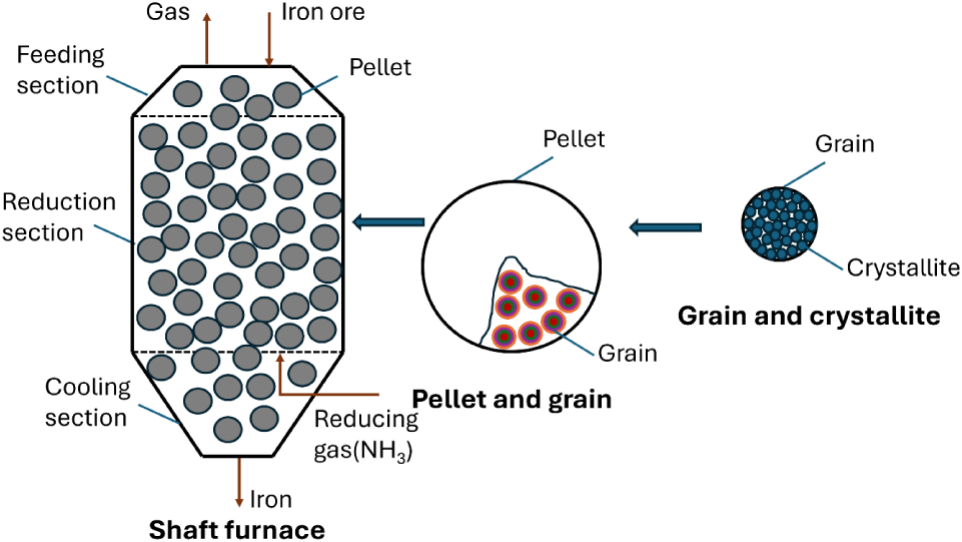
Structural depiction of the iron ore reduction process.

A 1-dimensional (1D) mathematical model of the shaft furnace is constructed with the following assumptions: (1) the shaft furnace is a large cylindrical vessel with wall heat loss; (2) the solid pellets are perfectly spherical and move downward with a constant velocity without breakage and size change; (3) the gases are assumed to follow the ideal gas law; (4) the gas and solid flows are distributed uniformly along the radial direction; and (5) the temperature of each phase is uniform along the radial direction. This work focuses on the kinetics of the reduction reactions and therefore adopts a simplified 1D plug flow model, which needs to be extended in the future to 2D or 3D simulations in order to capture future complexities pertaining to the multiphase flow behavior within the furnace.^35^ In real operation, the pellets may pulverize and crack in the furnace, leading to particles with reduced size, which could affect pressure drop as well as mass and heat transfer. This complexity has not been included in the first shaft furnace model for ADR. In addition, ideal gas behavior was assumed in the current model, which is a simplifying treatment and needs to be revised, particularly when the furnace operates at significantly elevated pressure.

### 2.2 Chemical Reaction Equations

From the previously reported experiments, it is known that the reduction by ammonia starts at 430 °C and under 570 °C, magnetite directly reduces to iron because wustite is thermodynamically unstable. Therefore, three zones of the reduction section of the furnace have been considered, divided by temperature level:^3,9,14^

In Zone 1 (<430 °C), no reaction occurs.

In Zone 2 (430–570 °C), the following reactions occur.

1 $$\frac{9}{2}Fe_{2}O_{3}+NH_{3}=3Fe_{3}O_{4}+\frac{1}{2}N_{2}+\frac{3}{2}H_{2}O$$

2 $$\frac{3}{8}Fe_{3}O_{4}+NH_{3}=\frac{9}{8}Fe+\frac{1}{2}N_{2}+\frac{3}{2}H_{2}O$$

3 $$3Fe_{2}O_{3}+H_{2}=2Fe_{3}O_{4}+H_{2}O$$

4 $$\frac{1}{4}Fe_{3}O_{4}+H_{2}=\frac{3}{4}Fe+H_{2}O$$

Zone 3 (>570 °C) involves most reduction reactions:

5 $$\frac{9}{2}Fe_{2}O_{3}+NH_{3}=3Fe_{3}O_{4}+\frac{1}{2}N_{2}+\frac{3}{2}H_{2}O$$

6 $$\frac{57}{32}Fe_{3}O_{4}+NH_{3}=\frac{45}{8}Fe_{0.95}O+\frac{1}{2}N_{2}+\frac{3}{2}H_{2}O$$

7 $$\frac{3}{2}Fe_{0.95}O+NH_{3}=\frac{57}{40}\mathrm{Fe}+\frac{1}{2}N_{2}+\frac{3}{2}H_{2}O$$

8 $$3Fe_{2}O_{3}+H_{2}=2Fe_{3}O_{4}+H_{2}O$$

9 $$\frac{19}{16}Fe_{3}O_{4}+H_{2}=\frac{15}{4}Fe_{0.95}O+H_{2}O$$

10 $$Fe_{0.95}O+H_{2}=\frac{19}{20}Fe+H_{2}O$$

Note that reduction reactions 1 and 3 are identical to 5 and 8, respectively. Additionally, ammonia decomposition occurs in zones 2 and 3 based on iron catalysts, as shown in reaction 11:

11 $$NH_{3}=\frac{1}{2}N_{2}+\frac{3}{2}H_{2}$$

Furthermore, the nitridation reactions occur in Zone 2 and Zone 3,^36,37^ as listed below:

12 $$4Fe+NH_{3}=Fe_{4}N+\frac{3}{2}H_{2}$$

13 $$3Fe_{4}N+NH_{3}=4Fe_{3}N+\frac{3}{2}H_{2},\left(T<700^{\;{}^{\circ}}C\right)$$

14 $$2Fe_{3}N+NH_{3}=3Fe_{2}N+\frac{3}{2}H_{2},\left(T<700^{{}^{\circ}}C\right)$$

15 $$Fe_{4}N=4Fe+\frac{1}{2}N_{2},\left(T>650^{\;{}^{\circ}}C\right)$$

### 2.3 Governing Equations

#### 2.3.1 Mass Balance Equations

The mass conservation equation for the gas-phase component *i* is

16 $$\varepsilon_{b}\frac{\partial C_{gi}}{\partial t}=-u_{g}\frac{\partial C_{gi}}{\partial z}+S_{gi}\left(z\right)$$

where *ε*_*b*_ is the bed voidage; *C*_*gi*_ is the concentration of gas; *u*_*g*_ is the gas superficial velocity; *S*_*gi*_ is the source term (per unit reactor volume); *t* is the time; *z* is the distance along the *z*-axis; *i* is the gas species.

The mass conservation equation for the solid phase component *j* is

17 $$\frac{\partial C_{sj}}{\partial t}=-u_{s}\frac{\partial C_{sj}}{\partial z}+S_{sj}\left(z\right)$$

where *C*_*sj*_ is the concentration of solid (per unit reactor volume); *u*_*s*_ is the solid bed moving velocity; *S*_*sj*_ is the source term of the solid phase (per unit reactor volume); *j* is the solid species.

#### 2.3.2 Heat Balance Equations

The energy equation for the gas phase is

18 $$\varepsilon_{b}C_{p,g}\frac{\partial T_{g}}{\partial t}=-u_{g}C_{p,g}\frac{\partial T_{g}}{\partial z}+\frac{\partial }{\partial z}\left(K_{g}\frac{\partial T_{g}}{\partial z}\right)+h_{gs}a_{gs}\left(T_{s}-T_{g}\right)+h_{gw}\frac{A_{w}}{V_{r}}\left(T_{Am}-T_{g}\right)$$

where *C*_*p,g*_ is the heat capacity of the gas phase; *T*_*g*_ is the temperature of the gas phase; *K*_*g*_ is the thermal conductivity of the gas phase; *h*_*gs*_ is the heat transfer coefficient between gas and solid; *a*_*gs*_ is the interfacial area between gas and solid (per unit reactor volume); *T*_*s*_ is the temperature of the solid phase; *T*_*Am*_ is the ambient temperature; *h*_*gw*_ is the wall heat transfer coefficient; *A*_*w*_ is the furnace wall area; *V*_*r*_ is the reactor volume.

The energy equation for the solid phase is

19 $$C_{p,s}\frac{\partial T_{s}}{\partial t}=-u_{s}C_{p,s}\frac{\partial T_{s}}{\partial z}+\frac{\partial }{\partial z}\left(K_{s}\frac{\partial T_{s}}{\partial z}\right)+h_{gs}a_{gs}\left(T_{g}-T_{s}\right)+Q_{r}$$

where *C*_*p,s*_ is the heat capacity of the solid phase; *Q*_*r*_ is the heat from reactions; *K*_*s*_ is the thermal conductivity of the solid phase. For details of heat-transfer parameters, please refer to Section S1 Modeling of Mass and Heat Transfer in the Supporting Information. (Here, the prefix “S” denotes Supporting Information. The Supporting Information is provided as a separate document and can be accessed via the link given at the end of this paper).

#### 2.3.3 Pressure Drop Equation

The gas flow pressure drop follows the Ergun equation:

20 $$\frac{\partial P}{\partial z}=f_{s}$$

21 $$f_{s}=-150\frac{{\left(1-\varepsilon_{b}\right)}^{2}}{{\varepsilon_{b}}^{3}}\frac{\mu_{g}}{{d_{p}}^{2}}u_{g}-1.75\frac{1-\varepsilon_{b}}{{\varepsilon_{b}}^{2}}\frac{\rho_{g}}{d_{p}}{u_{g}}^{2}$$

where *P* is the pressure; *f*_*s*_ is the source term; μ_*g*_ is the viscosity of gas; *d*_*p*_ is the pellet diameter; ρ_*g*_ is the gas density.

#### 2.3.4 Other Equations and Definitions

The change of gas flow rates along the furnace height due to the reactions is described by the following equation.

22 $$\frac{\partial F}{\partial z}=\frac{\pi }{4}{d_{r}}^{2}S_{g,total}$$

where *F* is the molar gas flow rate, mol/s; *d*_*r*_ is the reactor diameter; *S*_*g,total*_ is the gas phase source.

The gas superficial velocity, which varies along the furnace height, is thus determined by

23 $$u_{g}=\frac{F}{\frac{\pi }{4}{d_{r}}^{2}C_{gt}}$$

where *C*_*gt*_, gas phase total concentration, is in turn obtained from the ideal gas law:

24 $$C_{gt}=\frac{P}{RT_{g}}$$

### 2.4 Grain Model

In this work, the grain model is employed to describe the multiphase reactions, analogous to that previously adopted for HDR.^38^ An iron ore pellet consists of a large number of hematite grains. For the grain model, a core–shell structure is assumed. The core of a grain consists of unconverted hematite, and the shell layers represent successive reduced phases. The schematic diagram of the grain model is shown in Figure 1. The total conversion time includes the pellet external transfer time (*t*_*ext*_), pellet internal diffusion time (*t*_*dif*_) comprising intergranular diffusion time (*t*_*dif,p*_), intragranular diffusion time (*t*_*dif,gr*_), intracrystallite diffusion time (*t*_*dif,cr*_), and final chemical reaction time (*t*_*ch*_).^25,29,39^

The external gas transfer time (*t_ext_*) is

25 $$t_{ext}=\tau_{ext}X$$

where *τ_ext_* is the characteristic time for external gas transfer; *X* is the conversion ratio of solid.

The in-pellet gas diffusion time (*t*_*dif*_) is

26 $$t_{dif}=\tau_{dif}\left[1-3{\left(1-X\right)}^{2/3}+2\left(1-X\right)\right]$$

where τ_*dif*_ is the total characteristic time for gas diffusion, representing the sum of the characteristic times of three individual intrapellet diffusion steps introduced above.

The reaction time (*t*_*chem*_) is

27 $$t_{chem}=\tau_{chem}\left[1-{\left(1-X\right)}^{1/3}\right]$$

where τ_*chem*_ is the characteristic time for reaction.

The total diffusion time is

28 $$t=t_{ext}+t_{dif}+t_{chem}$$

29 $$\frac{dt}{dX}=\tau_{ext}+2\tau_{dif}\left({\left(1-X\right)}^{-\frac{1}{3}}-1\right)+\frac{\tau_{ch}}{3}{\left(1-X\right)}^{-\frac{2}{3}}$$

Thus, the solid conversion rate is

30 $$\frac{dX}{dt}={\left(\frac{dt}{dX}\right)}^{-1}$$

τ_*ext*_, τ_*dif*_, and τ_*chem*_ are derived based on the method in ref^38^. The details of characteristic times are shown in Section S2 Modeling of Characteristic Times and Table S1 in the Supporting Information.

The reaction rates for the reduction reaction of Fe_2_O_3_, Fe_3_O_4_, and Fe_0.95_O by H_2_ and NH_3_ are

31 $$r_{1,3,5,8}=\frac{1}{\upsilon_{i}}C_{Fe2O3}\frac{dX_{Fe2O3}}{dt}$$

32 $$r_{2,4,6,9}=\frac{1}{\upsilon_{i}}C_{Fe3O4}\frac{dX_{Fe3O4}}{dt}$$

33 $$r_{7,10}=\frac{1}{\upsilon_{i}}C_{FeO}\frac{dX_{FeO}}{dt}$$

where *i* is the reaction number (reactions (1)-(10)); ν_*i*_ the reaction coefficient (i.e., the stoichiometric coefficient of the corresponding iron oxide); and d*X*/d*t* is from eq 30.

The reaction rate (mol/s/kg (Catalyst)) for NH_3_ decomposition synthesis^40^ (reaction 11) is modeled as the negative rate of NH_3_ synthesis:^40^

34 $$r_{11}=-\frac{k_{2,0}\exp\left(-\frac{E_{A}}{RT}\right)\left({p_{N2}}^{0.5}K_{eq}{\left(\frac{{p_{H2}}^{1.5}}{p_{NH3}}\right)}^{\alpha }-{\left(\frac{p_{NH3}}{{p_{H2}}^{1.5}}\right)}^{1-\alpha }\right)}{1+K_{3}{p_{NH3}}^{f3}}$$

where *k*_2,0_ is the prefactor; *E*_*A*_ the activation energy; α, *K*_3_, *f*_3_ are coefficients.

The common nitrides in the process of ammonia reduction of iron ore are Fe_4_N, Fe_3_N, and Fe_2_N.^36^ The reaction rate for nitridation^41^ is

35 $$r_{12,13,14}=\frac{1}{\nu_{i}}C_{FexNy}k_{0}e^{-\frac{E_{a}}{RT}}\left(p_{NH3}-\frac{{p_{H2}}^{3/2}}{K_{eq,FexN}}\right)$$

where *v*_*i*_ is the stoichiometric coefficient, which is 4, 3, and 2 for reactions 12–14, respectively; *K*_*eq,FexN*_ is the equilibrium constant of nitridation reactions 12–14, which can be obtained from ref 41,42 . *p*_H2_ and *p*_NH3_ are the partial pressures (bar) of H_2_ and NH_3_; *k*_0_ is the prefactor (1/s), and *E*_*a*_ is the activation energy; Fe_*x*_N_*y*_ refers to Fe, Fe_4_N, Fe_3_N, or Fe_2_N.

The reaction rate for Fe_4_N decomposition, when the temperature is higher than 650 °C, is

36 $$r_{15}=k_{0}\exp\left(-\frac{E_{a}}{RT}\right)C_{Fe4N}$$

Further details of nitridation modeling are shown in Section S3 Further Details of Nitridation Modeling in the Supporting Information.

### 2.5 Reaction Rates for Individual Species

All of the reaction rates for three zones are summarized as follows:

Zone 1 (<430 °C):

37 $$R_{1,i}=0\;\mathrm{for each species}\;i\;\mathrm{or}\;j$$

Zone 2 (430–570 °C):

38 $$R_{2,Fe2O3}=-\frac{9}{2}r_{1}-3r_{3}$$

39 $$R_{2,Fe3O4}=3r_{1}-\frac{3}{8}r_{2}+2r_{3}-\frac{1}{4}r_{4}$$

40 $$R_{2,FeO}=0$$

41 $$R_{2,Fe}=\frac{9}{8}r_{2}+\frac{3}{4}r_{4}-4r_{12}+4r_{15}$$

42 $$R_{2,Fe4N}=r_{12}-3r_{13}-r_{15}$$

43 $$R_{2,Fe3N}=4r_{13}-2r_{14}$$

44 $$R_{2,Fe2N}=3r_{14}$$

45 $$R_{2,NH3}=-r_{1}-r_{2}-r_{11}-r_{12}-r_{13}-r_{14}$$

46 $$R_{2,H2}=-r_{3}-r_{4}+\frac{3}{2}r_{11}+\frac{3}{2}r_{12}+\frac{3}{2}r_{13}+\frac{3}{2}r_{14}$$

47 $$R_{2,H2O}=\frac{3}{2}r_{1}+\frac{3}{2}r_{2}+r_{3}+r_{4}$$

48 $$R_{2,N2}=\frac{1}{2}r_{1}+\frac{1}{2}r_{2}+\frac{1}{2}r_{11}+\frac{1}{2}r_{15}$$

Zone 3 (>570 °C):

49 $$R_{3,Fe2O3}=-\frac{9}{2}r_{5}-3r_{8}$$

50 $$R_{3,Fe3O4}=3r_{5}-\frac{57}{32}r_{6}+2r_{8}-\frac{19}{16}r_{9}$$

51 $$R_{3,FeO}=\frac{45}{8}r_{6}-\frac{3}{2}r_{7}+\frac{15}{4}r_{9}-r_{10}$$

52 $$R_{3,Fe}=\frac{57}{40}r_{7}+\frac{19}{20}r_{10}-4r_{12}+4r_{15}$$

53 $$R_{3,\mathrm{Fe4N}}=R_{2,\mathrm{Fe4N}};\;R_{3,\mathrm{Fe3N}}=R_{2,\mathrm{Fe3N}};\;R_{3,\mathrm{Fe2N}}=R_{2,\mathrm{Fe2N}}$$

54 $$R_{3,NH3}=-r_{5}-r_{6}-r_{7}-r_{11}-r_{12}-r_{13}-r_{14}$$

55 $$R_{3,H2}=-r_{8}-r_{9}-r_{10}+\frac{3}{2}r_{11}+\frac{3}{2}r_{12}+\frac{3}{2}r_{13}+\frac{3}{2}r_{14}$$

56 $$R_{3,H2O}=\frac{3}{2}r_{5}+\frac{3}{2}r_{6}+\frac{3}{2}r_{7}+r_{8}+r_{9}+r_{10}$$

57 $$R_{3,N2}=\frac{1}{2}r_{5}+\frac{1}{2}r_{6}+\frac{1}{2}r_{7}+\frac{1}{2}r_{11}+\frac{1}{2}r_{15}$$

In the above equations, *R*_*m*,*n*_ represents the net production rate of species *n* in zone *m*; *r*_*k*_ is the reaction rate of reaction *k*.

## 3 Model Implementation and Calibration

The software COMSOL 6.2 was employed to model the 1D plug-flow reduction process in the shaft furnace. The mass and energy conservation and pressure models were constructed using the template of the concentration form PDE (partial differential equation). The quintic method was used for discretization. The mesh had 1000 grids, and the resolver was SPOOLES nested dissection. The tolerance was physics-controlled.

The reaction kinetic parameters for iron ore reduction by hydrogen can be found from literature,^23,30,38^ but those for reduction by ammonia are not available. In order to resolve this problem, TGA experiments (TGA 5500, TA Instruments) were performed, with the experimental data used to estimate the unknown parameters. Therefore, the purpose of this section is to address the current lack of available kinetic parameters for direct ammonia reduction reactions; other kinetic parameters, including those for the nitridation reactions, were adopted from the literature, as described in Section S3 Further Details of Nitridation Modeling and Section S4 Further Details of Model Calibration and Kinetic Parameters in Supporting Information. The TGA experiments were carried out with different ammonia fractions (10% and 20% in a gas mixture of Ar and NH_3_) at temperatures of 600 and 800 °C for Zone 3 and with different temperatures (550 and 500 °C) at 20% ammonia in the gas mixture of Ar and NH_3_ for Zone 2. Ar is a carrier gas in TGA experiments and does not influence the kinetics of the reduction reactions other than affecting the partial pressure of ammonia, and our model considers other different gases (e.g., Ar, H_2_O, N_2_, H_2_) inclusively. For simulation, the hematite sample placed in the TGA crucible was treated as a pellet. The conditions used to simulate the TGA experiments are shown in Table S2 in Section S4 Further Details of Model Calibration and Kinetic Parameters of the Supporting Information. The kinetic parameter values calibrated against the TGA data, together with those obtained from the literature, are given in Table S3 in Section S4 Further Details of Model Calibration and Kinetic Parameters of the Supporting Information. The calibration to achieve the fitted reaction kinetic parameters is based on minimizing the deviation between experimentally measured changes in the mass of the solid sample and simulation results, which is calculated by

58 $$\delta_{d}=\frac{\overset{i=n}{\underset{i=1}{\sum }}\frac{\left|x_{exp}-x_{sim}\right|}{x_{exp}}}{n}$$

where δ_*d*_ is the deviation; *x*_*exp*_ is the experimental data; *x*_*sim*_ is the simulation data with the same time series as the experimental data collection; *n* is the total number of experimental data.

As shown in Figure 2, at 600 and 800 °C, the model-predicted curves are very close to the experimental curve with a mass loss of 70% and the deviations are less than 1.6%. At 500 and 550 °C, the model considers the nitridation reactions and the predicted curves approach the experimental data with deviations of less than 2.0%. The TGA experimental results show that the estimated reaction kinetic parameters accurately reproduce the experimental TGA curves under various operating conditions, with fitting deviations below 2.0%. Consequently, these calibrated kinetic parameters are considered sufficiently reliable for predicting the reaction behavior in the shaft furnace. Currently, no experimental data from an industrial ammonia-fed shaft furnace exists to validate our simulation results. However, our model is constructed using reaction kinetics, which are calibrated against data from TGA experiments.

**Figure 2:**
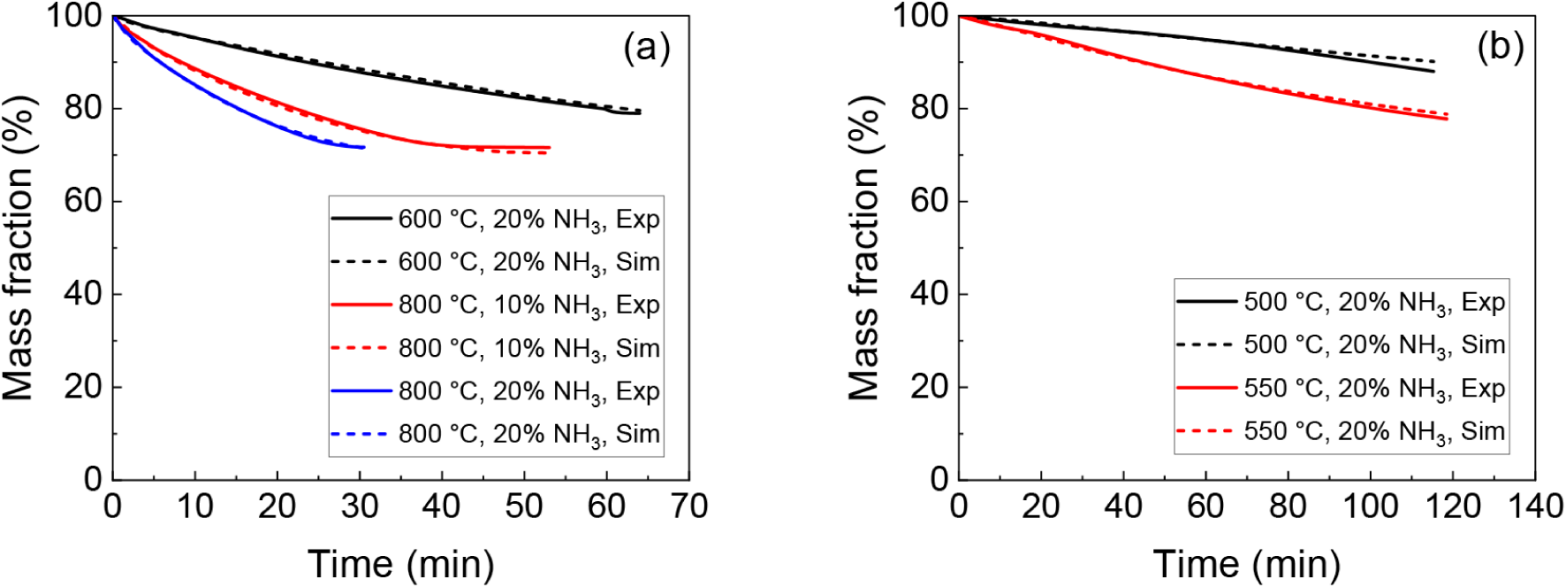
Comparison of TGA experiments and simulation results for Zone 3 (a) and Zone 2 (b) reactions.

## 4 Simulation Results and Discussion

In the simulations presented in this section, the default reactor settings are shown in Table 2, which hold for all simulation runs unless stated otherwise. The iron ore feeding rate corresponds approximately to the production of reduced iron at 32.08 kg/s or 1.0 × 10^6^ tons/year (including metallic iron and iron nitrides, with 8760 operational hours per year and 100% reduction). The influence of operational conditions is analyzed through variations from the default settings. Besides, the simulation results shown are all at 600 min, by which the process has reached a steady state.

**Table 2:** Default Conditions of Iron Ore Reduction by Ammonia Adopted in Shaft Furnace Simulations

Shaft furnace	Parameter	Value	Unit
Reactor	Height of furnace	5.0	M
	Diameter of furnace	5.5	M
	Bed porosity	0.5	-
Iron ore	Pellet size	1.5	Cm
	Grain size	25	μm
	Crystallite size	2	μm
	Pellet porosity	0.1	-
	Inter grain pore size	10	μm
	Intra grain pore size	0.5	μm
	Intra crystallite pore size	0.1	μm
	Inlet temperature	30	°C
	Inlet velocity	9.2 × 10^–4^	m/s
Gas	Inlet temperature	950	°C
	Inlet flow rate	3600	mol/s
	Inlet pressure	2.5	bar

The dimensions of the shaft furnace and operation conditions were based on the MIDREX furnace and ref^29^. The pellet structural parameters were adopted from ref^38^. The operation conditions satisfy the following two criteria: (1) gas velocity should be less than the minimum fluidization velocity; and (2) the water vapor content in the outlet gas mixture should be around 20% or lower, since excessive water vapor has been shown to impede the reduction of wüstite to metallic iron.^43^ The threshold of 20% outlet water vapor has also been reflected in existing simulation studies on hydrogen reduction of iron ores.^25,30^

### 4.1 Comparing ADR and HDR in a Shaft Furnace

First, to reveal how ADR behaves differently from HDR within a shaft furnace, we compared the simulation results of the two reduction processes under identical conditions (using the default settings shown in Table 2). Note that, consistent with previous studies,^26,28,30^ simulations of HDR ignored the unstable wustite at temperatures below 570 °C. The simulation results are shown in Figure 3. For hydrogen reduction, the main reactions occur in the upper section of the furnace. The final iron ore metallization rate, defined as the ratio of metallic iron concentration to the total iron concentration in the solid product, for HDR is 1.0, as shown in Figure 3a. For ammonia reduction, the reactions take place primarily in the middle section of the furnace, and the metallization rate is 0.85, as shown in Figure 3b. The significantly lower metallization rate of ADR is primarily caused by its nitridation reactions. Both processes clearly show the sequential steps of reduction from hematite, magnetite, and wustite to metallic iron along the furnace height. In the ADR furnace, the iron nitrides form at the low temperatures around 500–600 °C, but with the temperature increasing (i.e., moving downward along the furnace height, see Figure 3f), a portion of these nitrides decompose and the final nitridation rate, defined as the ratio of the concentration of iron in nitrides to the total iron concentration in the solid product, is 0.15.

**Figure 3:**
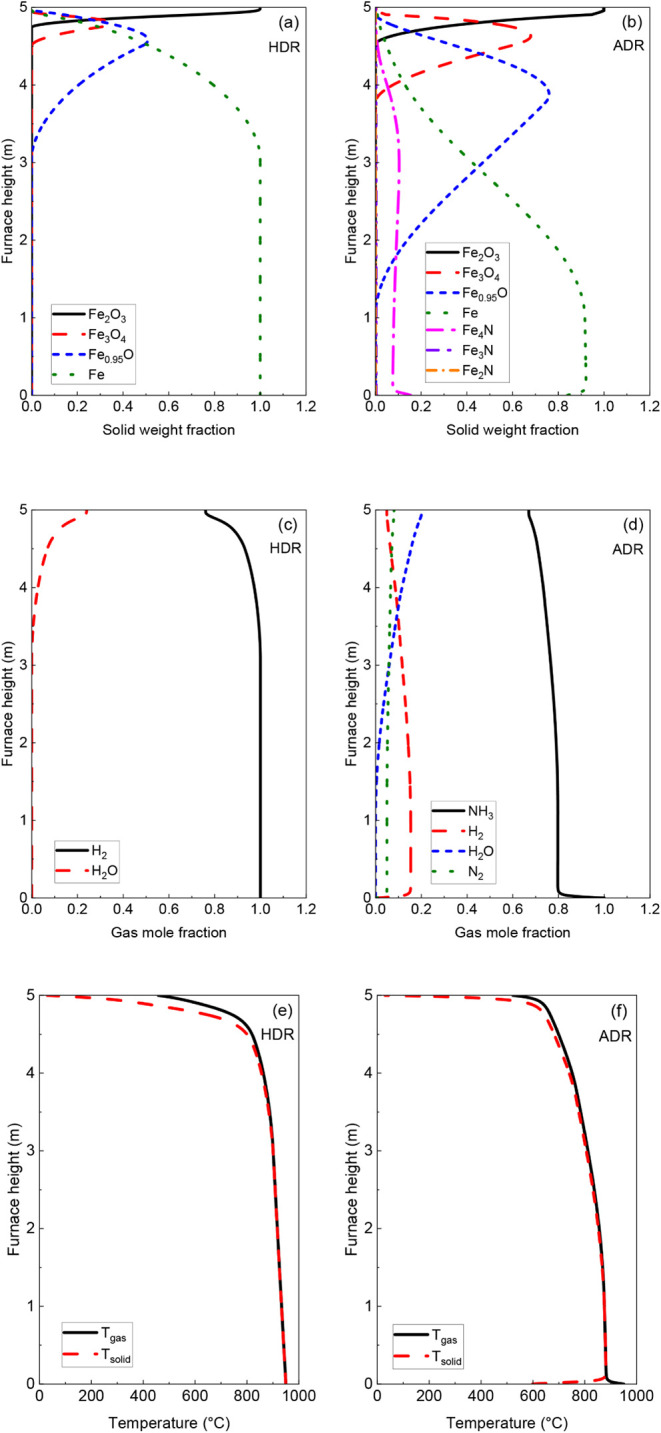
HDR and ADR shaft furnace simulation results: (a) solid fraction for HDR, (b) solid fraction for ADR, (c) gas fraction for HDR, (d) gas fraction for ADR, (e) temperature profile for HDR, and (f) temperature profile for ADR.

The gaseous product in the HDR furnace is only H_2_O, and the gas molar flow rate remains constant at 3600 mol/s. In contrast, the gaseous products in the ammonia furnace include H_2_, H_2_O, and N_2_, and the gas molar flow rate increases along the furnace height due to the reactions. As shown in Figure 3d, at the bottom of the ADR furnace, ammonia is partially decomposed into hydrogen and nitrogen with the reduced metallic iron as the catalyst. Therefore, the hydrogen fraction increases sharply in the near-bottom section, accompanied by a sharp decrease in the ammonia fraction. The hydrogen fraction gradually decreases along the ADR furnace height. The excess nitrogen gas in the system must be regularly removed to prevent its accumulation within the furnace if off-gases are recycled into the furnace to improve ammonia utilization (see Section 4.4).

The comparison of gas and solid temperatures in the furnaces is shown in Figure 3e and f. Because of the quick heat transfer in the moving bed, the solid temperature is generally very close to the gas temperature. For both HDR and ADR, the solid temperature in the furnace very quickly reaches above 400 °C from the feeding temperature. A distinct feature of the temperature profile of ADR is that the solid temperature drops sharply at the bottom of the furnace. The reason is that ammonia decomposition occurs in that area, which absorbs a significant amount of heat to sustain this strong endothermic reaction. The total transferred heat from gas to solid in the furnace is 8.35 × 10^10^ W for ammonia, which is 1.73 times higher than that for hydrogen reduction (4.84 × 10^10^ W). This contrast reflects the difference in the heat of reduction reactions between the two systems.

From the above results, some important characteristics of ADR of iron ore can be concluded in comparison with HDR: (1) the process of ADR absorbs a huge amount of heat and requires a higher heat supply; (2) the ammonia reduction process is more complex than HDR, including ammonia reduction reactions, ammonia decomposition into hydrogen, hydrogen reduction reactions, and possible further nitridation reactions; (3) the gaseous product from hydrogen reduction is only water vapor and is easy for postprocessing, while the gaseous products from ammonia reduction contain nitrogen, which needs to be removed from the system when gas recycling is needed.

### 4.2 Effect of Key Operating Conditions on ADR

Under the default reactor operating conditions, the final metallization rate of ADR reached only 0.85. To enhance metallization, the effects of gas inlet pressure, gas inlet temperature, gas inlet flow rate, and solid inlet velocity were investigated. The results are presented in Figure 4.

**Figure 4:**
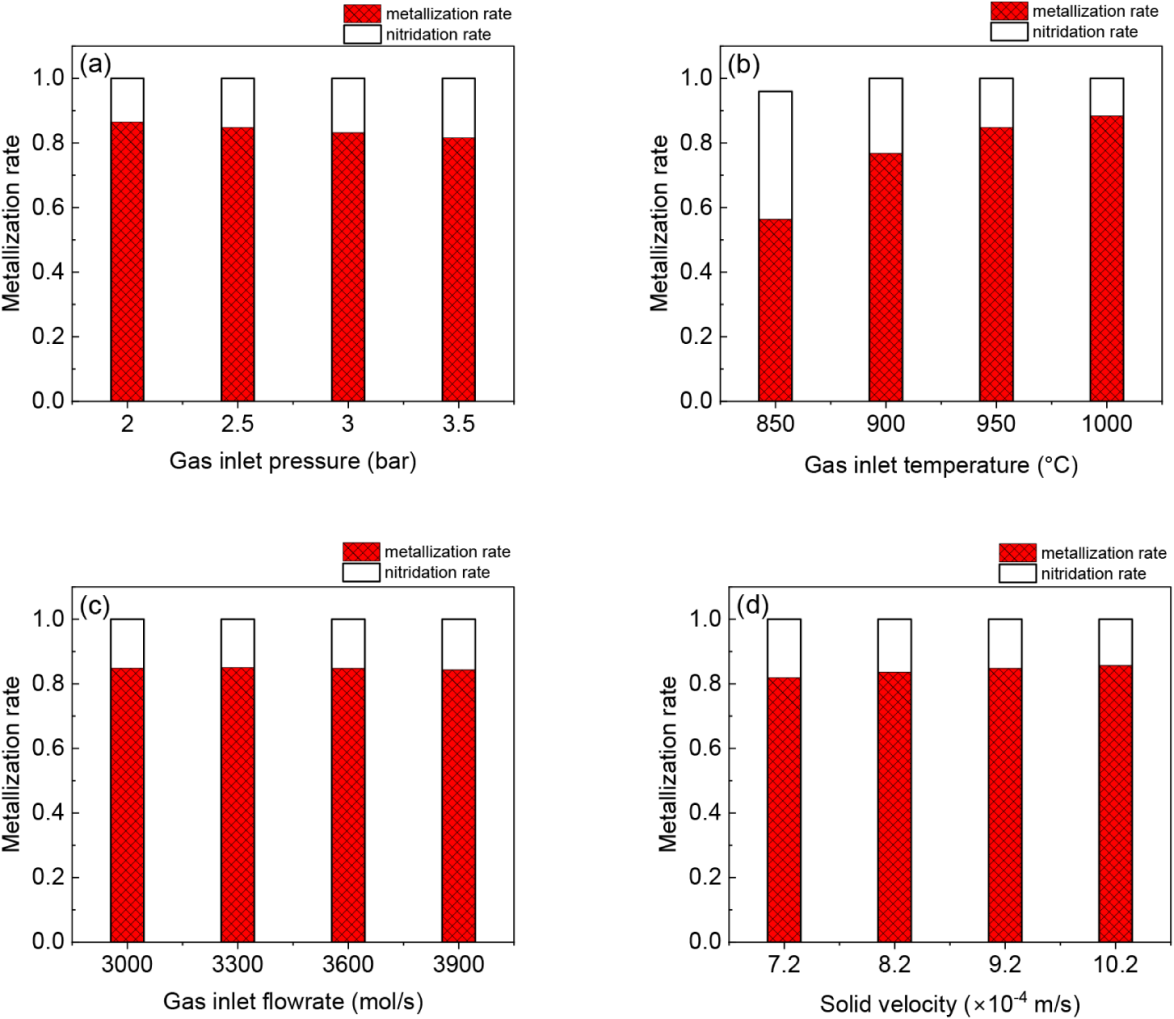
Effect of operation parameters: (a) gas inlet pressure, (b) gas inlet temperature, (c) gas inlet flow rate, and (d) solid velocity.

Figure 4a shows the effect of gas inlet pressure. All iron ore was reduced to iron, but a certain portion of iron was converted to iron nitrides. Increasing the pressure means increasing the gas concentration and decreasing the gas velocity. For example, when the pressure increases from 2.0 to 3.5 bar, the gas inlet velocity declines from 7.70 to 4.40 m/s. Therefore, the gas pressure is used to keep the gas velocity at or below the minimum fluidization velocity. Accompanying this increase of gas inlet pressure is a decrease in the metallization rate from 0.86 to 0.82 and an increase in the nitridation rate from 0.14 to 0.18, caused by higher ammonia concentration. Increasing the gas inlet temperature significantly enhances the metallization rate, as shown in Figure 4b. When the inlet temperature is raised from 850 to 1000 °C, the metallization rate increases from 0.56 to 0.88, while the total reduced iron ratio (including nitrides) is 0.96 for 850 °C or 1.0 for other temperatures. This demonstrates that the formation of Fe_4_N strongly depends on the temperature. Under higher temperatures (>650 °C), decomposition of Fe_4_N can commence, leading to a lower content in the final product. This has previously been demonstrated by Mat et al.^12^ with 40 wt % Fe_4_N or 21 atom % N in the iron at a temperature of 700 °C and by Park et al.^36^ with 1.2–7.6 wt % nitrogen under a temperature range of 600–850 °C. Over the same temperature range, the nitridation rate decreases significantly from 0.40 to 0.12. Increasing the gas inlet flow rate from 3000 to 3900 mol/s does not change the metallization rate and nitridation rate significantly, which remain approximately at 0.85 and 0.15, respectively, as shown in Figure 4c. This conclusion is valid for the tested range of gas flow rates between 3000 and 3900 mol/s, along with a gas inlet temperature of 950 °C, a gas inlet pressure of 2.5 bar, and a solid velocity of 9.2 × 10^–4^ m/s. Figure 4d shows the influence of solid velocity. As the solid velocity increases from 7.2 to 10.2 × 10^–4^ m/s, the metallization rate increases slightly from 0.82 to 0.86, while the nitridation rate declines from 0.18 to 0.14. A higher solid velocity corresponds to a shorter residence time, which inhibits nitridation.

From the above analysis, it is evident that under the studied conditions, a higher gas pressure, causing a reduced gas velocity, can slightly decrease the metallization. A higher gas inlet temperature increases the solid temperature, leading to increased metallization and reduced nitridation. The gas inlet flow rate and the solid velocity have a negligible effect on metallization and nitridation under the studied conditions. Among all investigated parameters, the gas inlet temperature is shown to be the most influential factor. Nitridation is strongly dependent on the solid temperature, with higher temperatures suppressing nitride formation. Overall, the solid temperature plays a critical role in determining the final metallization and nitridation outcomes of the reduction process.

### 4.3 Effect of Pellet Size

This section investigates the effects of pellet size on the final metallization rate. The results corresponding to a range of gas inlet temperatures are shown in Figure 5. Reducing the pellet size shortens the diffusion path length, thereby potentially enhancing the reduction process. As shown in Figure 5a, decreasing the pellet size from 15 to 6 mm increases the metallization rate from 0.56 (15 mm pellets at 850 °C) to 0.92 (6 mm pellets at a gas inlet temperature of 1000 °C). At a gas inlet temperature of 950 °C, the metallization rate increases from 0.85 to 0.88 as the pellet size decreases from 15 to 6 mm.

**Figure 5:**
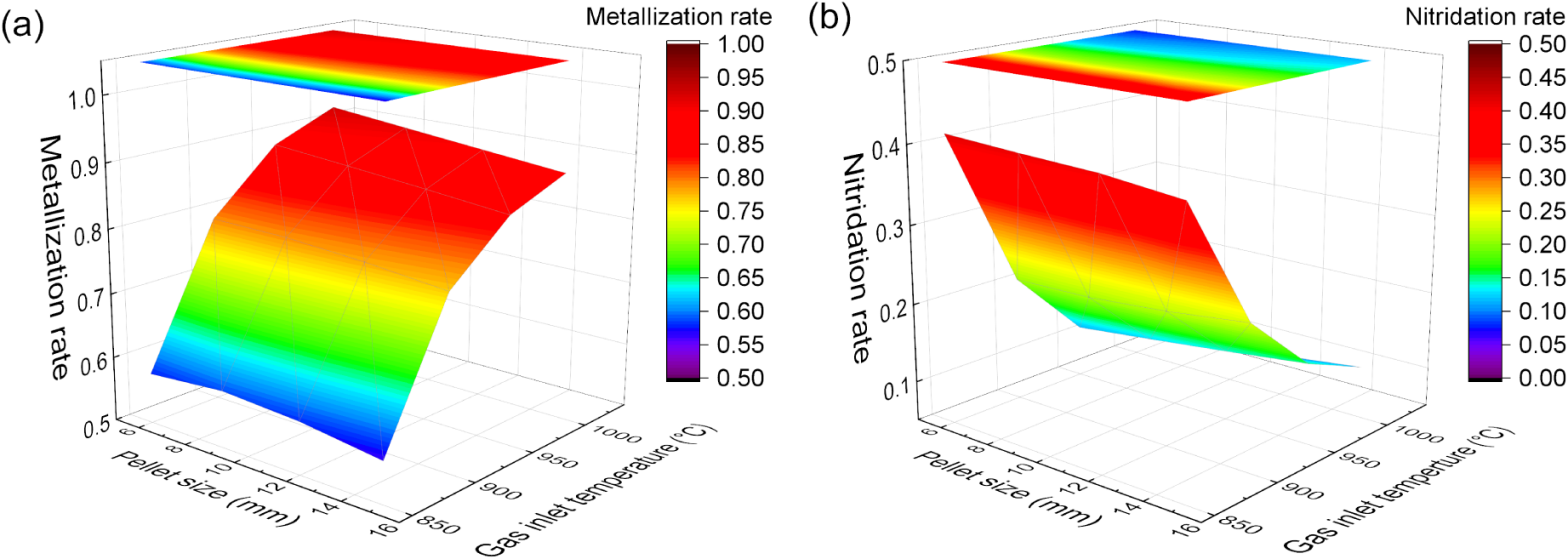
Effect of pellet size at different gas inlet temperatures: (a) metallization and (b) nitridation.

Figure 5b presents the nitridation rates under different pellet sizes and gas inlet temperatures. Lower temperatures and larger pellet sizes result in higher nitridation rates. Specifically, the nitridation rate increases from 0.08 to 0.42 as the pellet size increases from 6 to 15 mm and the gas inlet temperature decreases from 1000 to 850 °C. Overall, the pellet size and gas inlet temperature have a more pronounced impact on the reduction process than other investigated parameters.

### 4.4 Modeling Shaft Furnace with Gas Purging and Recycling

As mentioned earlier, ADR needs to deal with nitrogen removal from the system, since in real industrial operations, the exhaust gas of the shaft furnace needs to be recycled to maximize the utilization of NH_3_ (and H_2_ produced from NH_3_ decomposition). In addition to NH_3_ and H_2_, the exhaust gas flow contains nitrogen and water produced in the furnace, which need to be removed to avoid accumulation in the gas recycle loop. While water vapor removal could readily be implemented through condensation, removing nitrogen selectively through a separation process can be costly. The water vapor content in the outlet gas should be controlled to be around 20% because higher water vapor content blocks the reduction process.^37^ Here, we have designed a system that purges partially the produced outflow gases after reduction (with the purged gas to potentially provide a combustion fuel for process heating, not studied in this work; see below for a simulated purge ratio), removes part of the water vapor from the remaining gases (see below for a simulated water removal ratio), and finally returns the processed gases to the shaft furnace, as shown in Figure 6. Before reentering the shaft furnace, the processed gases are mixed with fresh ammonia and heated to 950 °C (the default inlet gas temperature). The flow rate of fresh ammonia (mol/s) needed is determined by the following equation:

59 $$F_{NH3\text{\_}fresh}=\frac{1}{3}\left(F_{H\text{\_}total}-2F_{H2\text{\_}recycle}-3F_{NH3\text{\_}recycle}\right)$$

where *F*_H*_total*_ represents the total molar flow rate of hydrogen atoms from H_2_ and NH_3_ in the furnace gas feed and is set to be identical to that corresponding to the default NH_3_ inlet flow rate (i.e., 3 × 3600 = 10800 mol/s); *F*_H2*_recycle*_, recycled H_2_ flow rate after purge (mol/s); *F*_NH3*_recycle*_, recycled NH_3_ flow rate after purge (mol/s). *F*_H2*_recycle*_ and *F*_NH3*_recycle*_ can be calculated from the following two equations:

60 $$F_{H2\text{\_}recycle}=F_{H2\text{\_}out}\left(1-r_{purge}\right)$$

61 $$F_{NH3\text{\_}recycle}=F_{NH3\text{\_}out}\left(1-r_{purge}\right)$$

where *F*_H2*_out*_ and *F*_NH3*_out*_ are the molar flow rates of H_2_ and NH_3_ in the outlet gas of the furnace; *r*_*purge*_ is the purge ratio.

**Figure 6:**
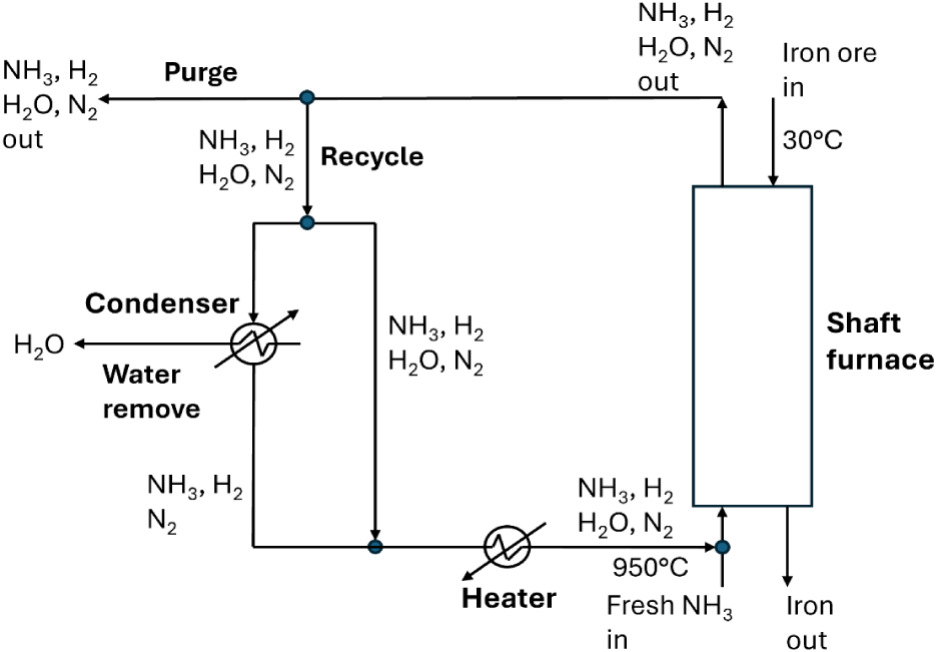
Schematic diagram of the shaft furnace with gas recycling.

With the purge ratio and the water removal ratio set to 0.3 and 0.8, respectively, the simulation was able to reach a steady state after 550 min. The gas pressure was set to 3.2 bar to keep the gas velocity lower than the minimum fluidization velocity. This design is able to maintain the steady state and satisfactory operation of the system without the need for selective removal of nitrogen from the recycled stream; the simulation results are shown in Figure 7. The final metallization rate of 0.92, combined with a nitridation rate of 0.08, yields a total reduction degree of 1.0, as shown in Figure 7a. As illustrated in Figure 7b, the stable inlet flow rates of NH_3_, H_2_, H_2_O, and N_2_ are 3530.9, 100.9, 140.9, and 692.9 mol/s, respectively, and the total flow rate for steady state is 4465.6 mol/s, increasing from 3600 mol/s of NH_3_ in the case without gas recycling and from the initial operating 3600 mol/s with gas recycling. The inlet water vapor content is 3.2%, which is less than 10–14% suggested in the literature,^43,44^ and the outlet water vapor content is 20%, a level that is not expected to hinder the reduction process (as mentioned earlier). As shown in Figure 7c, the solids throughout the furnace are heated rapidly, and the solid temperature near the furnace bottom approaches the gas temperature. Finally, despite the increased gas flow rate through gas recycling, Figure 7d shows that the gas velocity (5.8–6.7 m/s) remains lower than the minimum fluidization velocity (6.3–7.1 m/s, estimated from Wen–Yu correlation^45^).

**Figure 7:**
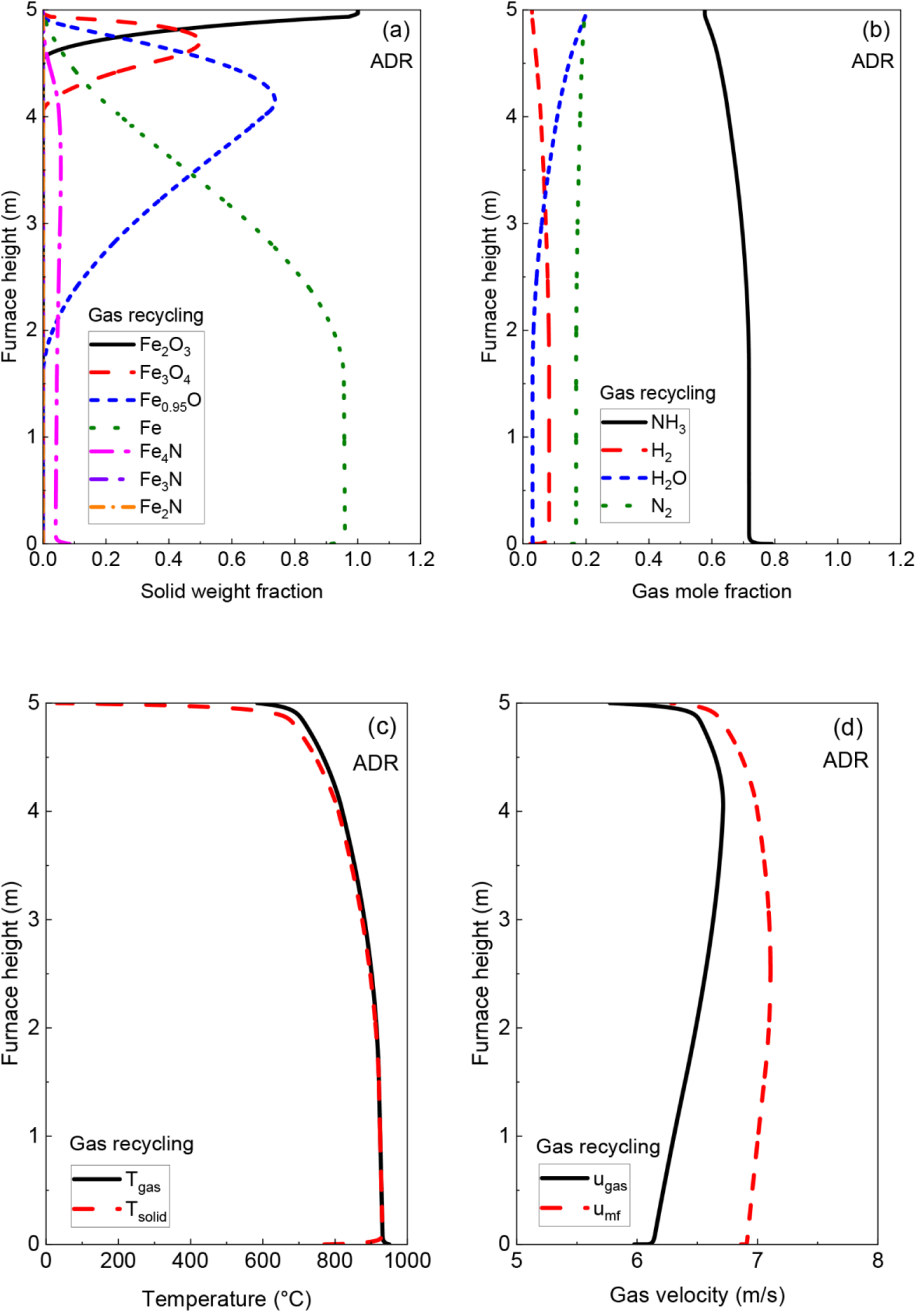
Shaft furnace steady-state results with gas recycling (NH_3_, *r_purge_* = 0.3, *r*_H2O*remove*_ = 0.8): (a) solid fraction, (b) gas fraction, (c) temperature profile, and (d) gas velocity.

Altogether, the above results demonstrate that the recycle design can control the accumulation of nitrogen gas and water vapor to realize a steady-state operation of the ADR furnace, which, compared to the system without gas recycling (Figure 3b,d,f), improves heat supply and achieves a desirable metallization rate.

To investigate the effects of purge ratio and water removal ratio on metallization, water vapor content, solid exit temperature, and gas inlet velocity, a series of simulations were conducted. The gas inlet pressure was set to 4.6 bar to retain the gas flow velocity in the fixed bed range. The results are listed in Figure 8. The metallization rate increases with decreasing purge ratio and water removal ratio, exceeding 0.90 when the purge ratio is below 0.2, as shown in Figure 8a. This is due to the reduction in the nitridation rate arising from an increased solid temperature, which (as shown in Figure 8c) is in turn caused by the increase in the total gas flow rate in the furnace when lower purge and water removal ratios are adopted (as shown in Figure 8d). Figure 8b indicates that the outlet water vapor content increases with decreasing purge ratio and water removal ratio in the range of 0.19 to 0.35, which undesirably exceeds the previously introduced 20% threshold.

**Figure 8:**
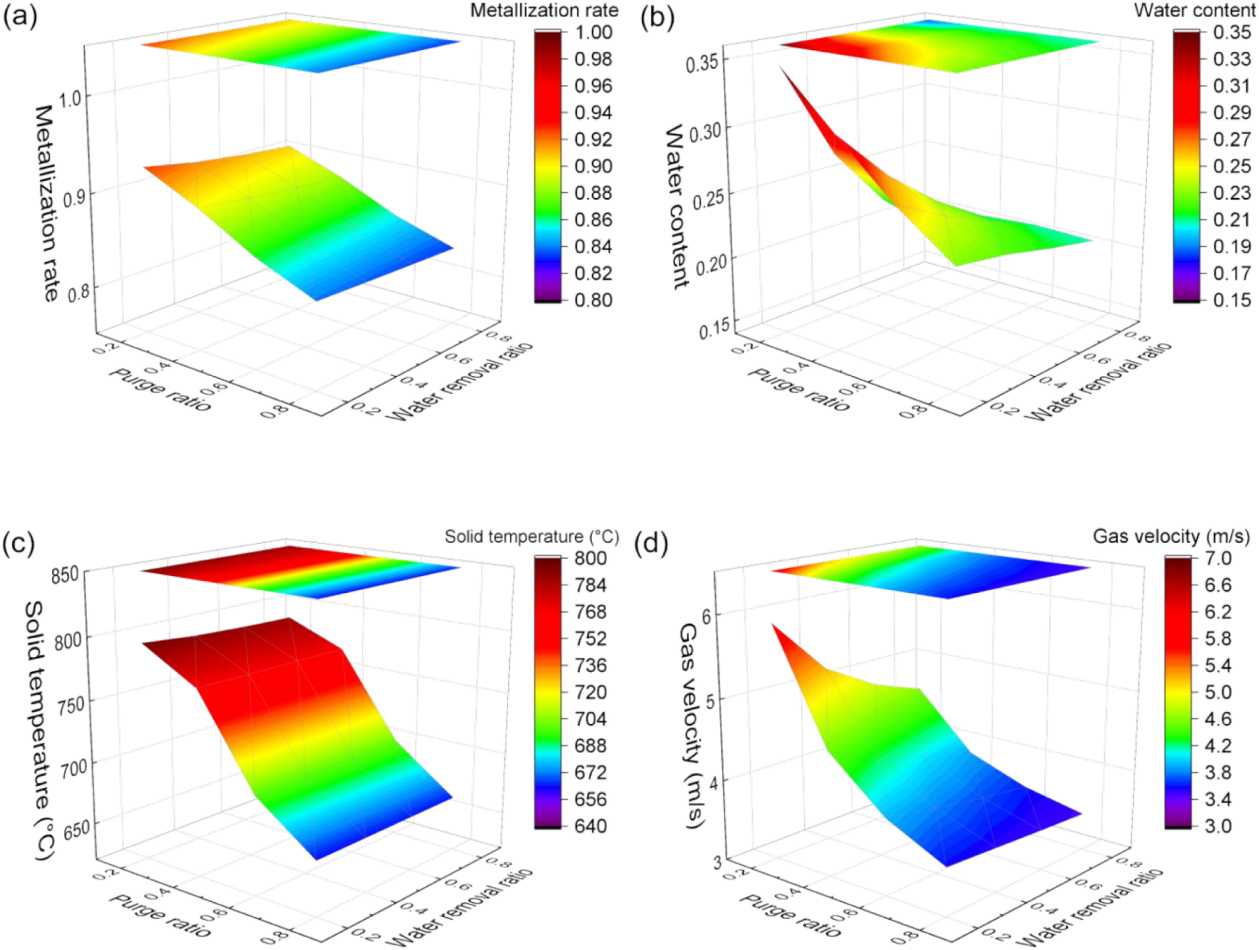
Effect of purge ratio and water removal ratio: (a) metallization rate, (b) water content, (c) solid outlet temperature, and (d) gas inlet velocity.

These results indicate that the choice of purge and water removal ratios will need to consider the trade-off between the different aspects of the process, particularly the metallization rate and water vapor content control.

## 5 Conclusions

This work has developed a 1D model to systematically evaluate the physical and chemical behaviors of ammonia direct reduction (ADR) in the shaft furnace. It has been found:

Compared to hydrogen, ammonia reduction of iron ore represents a more complex process that involves ammonia decomposition, ammonia and hydrogen direct reduction, and nitridation. This complexity particularly affects the thermal behavior of the furnace, and the highly endothermic ammonia decomposition results in a significant drop in solid temperature at the bottom of the furnace.
Inlet gas temperature stands out as the most influential parameter: a higher inlet gas temperature leads to more effective heating of the solid and an improved metallization rate. The pressure and flow rate of the inlet gas, solid feed velocity, and pellet size affect the performance of the furnace as well.
Unlike hydrogen, the metallization rate of ammonia reduction can be significantly affected by nitridation. While the iron ore reduction rate can reach 100%, the nitridation rate could reach nearly 50%, particularly when the inlet gas temperature reduces from ∼950 °C (where nitridation is negligible) to 850 °C. Higher ammonia concentrations in the inlet gas can also contribute to elevated nitridation.
Nitrogen gas is produced in the ADR furnace, which cannot be as easily separated as water vapor. The simulation shows that, by purging the recycled gas stream, coupled with water condensation, the shaft furnace can operate stably with a satisfactory metallization rate. This limits the water vapor in the gas in the furnace to below 20%, which avoids hindering the reduction process.

Overall, this work can guide subsequent research and design of the ADR shaft furnace through a mathematical representation of the coupled mass transfer, heat transfer, and reaction characteristics of the sophisticated process. It can also form the basis for wider modeling studies needed to facilitate a more comprehensive comparison between hydrogen and ammonia for green ironmaking.

## Supplementary Material



## Notes

Section S1: equations for modeling mass and heat transfer; Section S2: equations for modeling characteristic times and the expressions of characteristic times; Section S3: further details of nitridation modeling; Section S4: further details of model calibration by TGA experiments and kinetic parameters (PDF)
