# Supplementary material for: Modeling and Assessment of Ammonia Direct Reduction for Decarbonizing Iron Production
Source: ACS Omega. 2026 Jul 10;11(28):42610–22. doi: 10.1021/acsomega.6c03735 (PMC13393371; doi:10.1021/acsomega.6c03735)
Supplement: Supplementary file 1 [file ao6c03735_si_001.pdf]

Supporting Information

Modeling and Assessment of Ammonia Direct Reduction for Decarbonizing Iron Production

Xuesong Lu, Dorcas Tuitoek, Binjian Nie, Aidong Yang**

Department of Engineering Science, University of Oxford, Parks Road, Oxford OX1 3PJ, UK

S1 MODELING OF MASS AND HEAT TRANSFER

The external transfer coefficient ($k_{ext,i}$)[S1] used for gas component *i* is

$$k_{ext,i} = \frac{D_i Sh_i}{d_p} \tag{S1}$$

$$Sh_i = 2 + 1.1Re^{0.6}Sc_i^{\frac{1}{3}} \tag{S2}$$

where D_i is the diffusion coefficient; *Sh*, Sherwood number; *Re*, Reynolds number; *Sc*, Schmidt number; *i*, gas species.

The effective diffusion coefficient of gas component *i* in inter and intra grains[S2] is

$$D_{eff} = \frac{\varepsilon}{\tau}\left(\frac{1}{D_{Kn}} + \frac{1}{D_i}\right)^{-1} \tag{S3}$$

$$D_{Kn} = \frac{1}{3} d_{pore}\left(\frac{8RT_g}{\pi M_w}\right)^{\frac{1}{2}} \tag{S4}$$

where D_{eff} is the effective diffusion coefficient; ε, porosity; τ, tortuosity; d_{pore}, pore size; M_w, molecular weight; T_g, gas temperature.

The effective diffusion coefficient for intra crystals[S2] is

$$D_{eff} = \frac{\varepsilon}{\tau} D_{Kn\_ini} e^{-\frac{3t}{\tau_f}} + \left(1 - \varepsilon_{p\_ini} e^{-\frac{2t}{\tau_f}}\right) D_i \quad \text{(S5)}$$

where t refers to residence time of solids; τ_f is the sintering time factor, based on Reference (S2).

The gas-solid transferred heat (Q_{gs}) is obtained from the following equation:

$$Q_{gs} = a_{gs} h_{gs} \left(T_g - T_s\right) \quad \text{(S6)}$$

where T_s and T_g are the solid and gas temperatures; a_{gs}, the interfacial area; h_{gs}, heat transfer coefficient.

The wall heat loss is

$$Q_{gw} = h_{gw} \frac{A_w}{V_g} \left(T_{am} - T_g\right) \quad \text{(S7)}$$

where h_{gw} is heat transfer coefficient for the furnace wall, 100 $W/(m^2K)$[S3]; A_w, surface area of the furnace wall; V_g, volume of the gas in the furnace; T_{am}, ambient temperature.

The interfacial area between gas and solid (per unit rector volume) is

$$a_{gs} = \frac{6(1-\varepsilon_b)}{d_p} \quad \text{(S8)}$$

where ε_b is the bed porosity; d_p, pellet diameter.

The heat transfer coefficient between gas and solid is calculated from the following correlation.[S4]

$$h_{gs} = \frac{K_g}{d_p} \left(2 + 0.6 \left(\frac{Re}{\varepsilon_b}\right)^{\frac{1}{2}} Pr^{\frac{1}{3}}\right) \quad \text{(S9)}$$

where K_g is the heat conductivity of gas; $Re,$ Reynold number; Pr, Prandtl number.

The effective thermal conductivity for pellet[S5] is

$$K_{s,eff} = \frac{\varepsilon_p K_g + (1-\varepsilon_p) K_s}{3} + \frac{2}{3\left(\frac{\varepsilon_p}{K_g} + \frac{1-\varepsilon_p}{K_s}\right)} \quad \text{(S10)}$$

where K_s is the heat conductivity of solid; ε_p, pellet porosity.

S2 MODELING OF CHARACTERISTIC TIMES

Table S1 lists all the characteristic times for H_2 and NH_3. The derivation is referred to References (S2) and (S6). In Table S1, k_{ch} is the forward reaction rate:

$$k_{ch} = k_0 e^{-\frac{E_a}{RT}} \tag{S11}$$

where k_0 is the pre-exponential factor, E_a is the apparent activation energy. The activation energy values for ammonia reduction reactions haven't been reported previously and are estimated in this work from the calibration by TGA experimental results.

All the thermodynamic data on the species of enthalpy, entropy and heat capacity are from the NIST Chemistry WebBook.[S7] Estimation of viscosity and conductivity is based on Sutherland's formula. Heat capacities of Fe_4N and enthalpies of Fe_4N are from References (S8) and (S9). Reaction heat is estimated from the formation enthalpy of reactants and products. Density, viscosity and heat capacity of gas mixture are estimated from the following equation:

$$\left(\rho, \mu, C_p, K\right)_{g,mixture} = \sum_{i=1}^{n} x_i \left(\rho, \mu, C_p, K\right)_{g,i} \tag{S12}$$

where ρ is the density; μ, viscosity; C_p, the heat capacity; λ, conductivity; x_i, gas species fraction; i, gas species.

The equilibrium fractions of H_2 and NH_3 with respect to individual reduction reactions are

$$X_{H2,eq} = \frac{\left(1-y_{inert\_H2}\right)}{\left(1+K_{eq,H2}\right)} \tag{S13}$$

$$X_{NH3,eq} = \frac{\left(0.64952\left(1-y_{inert\_NH3}\right)+\frac{K_{eq,NH3}}{C_{gt}}\right)-\sqrt{\left(0.64952\left(1-y_{inert\_NH3}\right)+\frac{K_{eq,NH3}}{C_{gt}}\right)^2-0.42187\left(1-y_{inert\_NH3}\right)^2}}{0.64952} \tag{S14}$$

$$K_{eq} = \frac{1[bar]}{RT} e^{\left(-\frac{\Delta G}{RT}\right)} \tag{S15}$$

$$K_{eq} = e^{\left(-\frac{\Delta G}{RT}\right)} \tag{S16}$$

where y_{inert_H2} and y_{inert_NH3} is the fraction of inert gas, $y_{inert\_H2} = x_{Ar} + x_{NH3} + x_{N2}$, $y_{inert\_NH3} = x_{Ar} + x_{H2}$, x_i fraction of gas species; ΔG, Gibbs free energy of reaction; R, gas constant.

Table S1. Expression of the characteristic times.

	External transfer	Inter-granular diffusion	Intra-granular diffusion	Intra-crystallite diffusion with sintering	Chemical reaction
Zone 1	0	0	0	0	0
Zone2: Hematite → magnetite	$\tau_{ext1,H_2} = \frac{C_{Fe_2O_3,in,p} d_p}{18 k_{ext,H_2} C_{gt} (x_{H_2} - X_{H_2,eq})}$ $\tau_{ext1,NH_3} = \frac{C_{Fe_2O_3,in,p} d_p}{27 k_{ext,NH_3} C_{gt} (x_{NH_3} - X_{NH_3,eq})}$	$\tau_{dif1,p,H_2} = \frac{C_{Fe_2O_3,in,p} {d_p}^2}{72 D_{H_2,eff,p} C_{gt} (x_{H_2} - X_{H_2,eq})}$ $\tau_{dif1,p,NH_3} = \frac{C_{Fe_2O_3,in,p} {d_p}^2}{108 D_{NH_3,eff,p} C_{gt} (x_{NH_3} - X_{NH_3,eq})}$			$\tau_{chem1,H_2} = \frac{C_{Fe_2O_3,in,gr} d_{gr}}{6 k_{ch1,H_2} C_{gt} \left(x_{H_2} - \frac{x_{H_2O}}{K_{eq,H_2}}\right)}$ $\tau_{chem1,NH_3} = \frac{C_{Fe_2O_3,gcr} d_{gcr}}{9 k_{ch1,NH_3} C_{gt} \left(x_{NH_3} - C_{gt} \frac{{x_{N_2}}^{\frac{1}{2}} {x_{H_2O}}^{\frac{3}{2}}}{K_{eq,NH_3}}\right)}$
Zone2: Magnetite → iron	$\tau_{ext2,H_2} = \frac{2 C_{Fe_3O_4,in,p} d_p}{3 k_{ext,H_2} C_{gt} (x_{H_2} - X_{H_2,eq})}$ $\tau_{ext2,NH3} = \frac{4 C_{Fe_3O_4,in,p} d_p}{9 k_{ext,NH_3} C_{gt} (x_{NH_3} - X_{NH_3,eq})}$	$\tau_{dif2,p,H_2} = \frac{C_{Fe_3O_4,in,p} {d_p}^2}{6 D_{H_2,eff,p} C_{gt} (x_{H_2} - X_{H_2,eq})}$ $\tau_{dif2,p,NH_3} = \frac{C_{Fe_3O_4,in,p} {d_p}^2}{9 D_{NH3,eff,p} C_{gt} (x_{NH_3} - X_{NH_3,eq})}$	$\tau_{dif,gr,H_2} = \frac{C_{Fe_3O_4,gr} {d_{gr}}^2}{6 D_{H_2,eff,gr} C_{gt} (x_{H_2} - X_{H_2,eq})}$ $\tau_{dif,gr,NH_3} = \frac{C_{Fe_3O_4,gr} {d_{gr}}^2}{9 D_{NH_3,eff,gr} C_{gt} (x_{NH_3} - X_{NH_3,eq})}$		$\tau_{chem2,H_2} = \frac{C_{Fe_3O_4,in,gr} d_{gr}}{\frac{1}{2} k_{ch2,H_2} C_{gt} \left(x_{H_2} - \frac{x_{H_2O}}{K_{eq,H_2}}\right)}$ $\tau_{chem2,NH_3} = \frac{C_{Fe_3O_4,in,gr} d_{gr}}{\frac{3}{4} k_{ch2,NH_3} C_{gt} \left(x_{NH_3} - C_{gt} \frac{{x_{N_2}}^{\frac{1}{2}} {x_{H_2O}}^{\frac{3}{2}}}{K_{eq,NH_3}}\right)}$
Zone3: Hematite → magnetite	$\tau_{ext1,H_2} = \frac{C_{Fe_2O_3,in,p} d_p}{18 k_{ext,H_2} C_{gt} (x_{H_2} - X_{H_2,eq})}$ $\tau_{ext1,NH_3} = \frac{C_{Fe_2O_3,in,p} d_p}{27 k_{ext,NH_3} C_{gt} (x_{NH_3} - X_{NH_3,eq})}$	$\tau_{dif1,p,H_2} = \frac{C_{Fe_2O_3,in,p} {d_p}^2}{72 D_{H_2,eff,p} C_{gt} (x_{H_2} - X_{H_2,eq})}$ $\tau_{dif1,p,NH_3} = \frac{C_{Fe_2O_3,in,p} {d_p}^2}{108 D_{NH3,eff,p} C_{gt} (x_{NH_3} - X_{NH_3,eq})}$			$\tau_{chem1,H_2} = \frac{C_{Fe_2O_3,in,gr} d_{gr}}{6 k_{ch1,H_2} C_{gt} \left(x_{H_2} - \frac{x_{H_2O}}{K_{eq,H_2}}\right)}$ $\tau_{chem1,NH_3} = \frac{C_{Fe_2O_3,in,gr} d_{gr}}{9 k_{ch1,NH_3} C_{gt} \left(x_{NH_3} - C_{gt} \frac{{x_{N_2}}^{\frac{1}{2}} {x_{H_2O}}^{\frac{3}{2}}}{K_{eq,NH_3}}\right)}$
Zone3: Magnetite → wustite	$\tau_{ext2,H_2} = \frac{8 C_{Fe_3O_4,in,p} d_p}{57 k_{ext,H_2} C_{gt} (x_{H_2} - X_{H_2,eq})}$ $\tau_{ext2,NH_3} = \frac{16 C_{Fe_3O_4,in,p} d_p}{171 k_{ext,NH_3} C_{gt} (x_{NH_3} - X_{NH_3,eq})}$	$\tau_{dif2,p,H_2} = \frac{2 C_{Fe_3O_4,in,p} {d_p}^2}{57 D_{H_2,eff,p} C_{gt} (x_{H_2} - X_{H_2,eq})}$ $\tau_{dif2,p,NH3} = \frac{4 C_{Fe_3O_4,in,p} {d_p}^2}{171 D_{NH_3,eff,p} C_{gt} (x_{NH_3} - X_{NH_3,eq})}$			$\tau_{chem2,H_2} = \frac{8 C_{Fe_3O_4,in,gr} d_{gr}}{19 k_{ch2,H_2} C_{gt} \left(x_{H_2} - \frac{x_{H_2O}}{K_{eq,H_2}}\right)}$ $\tau_{chem2,NH_3} = \frac{16 C_{Fe_3O_4,in,gr} d_{gr}}{57 k_{ch2,NH_3} C_{gt} \left(x_{NH_3} - C_{gt} \frac{{x_{N_2}}^{\frac{1}{2}} {x_{H_2O}}^{\frac{3}{2}}}{K_{eq,NH_3}}\right)}$
Zone3: Wustite → iron	$\tau_{ext3,H_2} = \frac{C_{Fe_{0.95}O,in,p} d_p}{6 k_{ext,H_2} C_{gt} (x_{H_2} - X_{H_2,eq})}$	$\tau_{dif3,p,H_2} = \frac{C_{Fe_{0.95}O,in,p} {d_p}^2}{24 D_{H_2,eff,p} C_{gt} (x_{H_2} - X_{H_2,eq})}$	$\tau_{dif,gr,H_2} = \frac{C_{Fe_{0.95}O,in,gr} {d_{gr}}^2}{24 D_{H_2,eff,gr} C_{gt} (x_{H_2} - X_{H_2,eq})}$	$\tau_{dif,cr,H_2} = \frac{C_{Fe_{0.95}O,in,cr} {d_{cr}}^2}{24 D_{H_2,eff,cr} C_{gt} (x_{H_2} - X_{H_2,eq})}$	$\tau_{chem3,H_2} = \frac{C_{Fe_{0.95}O,in,cr} d_{cr}}{2 k_{ch3,H_2} C_{gt} \left(x_{H_2} - \frac{x_{H_2O}}{K_{eq,H_2}}\right)}$

	$\tau_{ext3,NH_3} = \frac{C_{Fe_{0.95}O,in,p} d_p}{18 k_{ext,NH_3} C_{gt} \left(x_{NH_3} - X_{NH_3,eq}\right)}$	$\tau_{dif3,p,NH_3} = \frac{C_{Fe_{0.95}O,in,p} {d_p}^2}{36 D_{NH_3,eff,p} C_{gt} \left(x_{NH_3} - X_{NH_3,eq}\right)}$	$\tau_{dif,gr,NH_3} = \frac{C_{Fe_{0.95}O,in,gr} {d_{gr}}^2}{36 D_{NH_3,eff,gr} C_{gt} \left(x_{NH_3} - X_{NH_3,eq}\right)}$	$\tau_{dif,cr,NH_3} = \frac{C_{Fe_{0.95}O,in,cr} {d_{cr}}^2}{36 D_{NH_3,eff,cr} C_{gt} \left(x_{NH_3} - X_{NH_3,eq}\right)}$	$\tau_{chem3,NH_3} = \frac{C_{Fe_{0.95}O,in,cr} d_{cr}}{3 k_{ch3,NH_3} C_{gt} \left(x_{NH_3} - C_{gt} \frac{{x_{N_2}}^{\frac{1}{2}} {x_{H_2O}}^{\frac{3}{2}}}{K_{eq,NH_3}}\right)}$

S3 FURTHER DETAILS OF NITRIDATION MODELING

The activation energies from Fe to Fe_4N, Fe_4N to Fe_3N, Fe_3N to Fe_2N are 38, 43 and 100 kJ/mol, correspondingly.[S10] The pre-factor, k_0 can be derived from Figure 6 in Reference (S10).

From Fe to Fe_4N, the equilibrium constant[11] for reaction (12) as a function temperature T (in K) is

$$2logK_{P1} = -\frac{2305}{T} + 3.2676logT + 0.0021174T - 3.72 \times 10^{-7}T^2 - 6.8 \quad \text{(S17)}$$

From Fe_4N to Fe_2N, the combined equilibrium constant[S11] for reaction (13) and (14) is

$$2logK_{P2} = -\frac{3548}{T} + 3.2676logT + 0.0021174T - 3.72 \times 10^{-7}T^2 - 6.8 \quad \text{(S18)}$$

From reaction stoichiometry, the equilibrium constants from Fe_4N to Fe_3N and Fe_3N to Fe_2N are the cube root and square root of the equilibrium constants from Fe_4N to Fe_2N, respectively. Note that K_{p1} and K_{p2} are based on the pressure unit of atm.

S4 FURTHER DETAILS OF MODEL CALIBRATION AND KINETIC PARAMETERS

The simulation conditions for TGA experiments are shown in Table S2. The reaction kinetic parameters for ammonia reduction of iron ore are shown in Table S3.

Table S2. Simulation conditions of TGA experiments.

TGA	Parameters	Value	Unit
Furnace	Assumed effective height of tube	7	mm
	Diameter of tube	18	mm
	Bed porosity	0.1	-
Fe_2O_3 (hematite)	Solid loading	50-60	mg

	Crucible diameter	6.7	mm
	Crucible height	5	mm
	Pellet porosity	0.35	-
	Inlet Temperature	500, 550, 800	°C
	Inlet velocity	0	m/s
Gas (NH_3/Ar)	Inlet temperature	500, 550, 800	°C
	Inlet flowrate	60	ml/min
	Inlet pressure	1.1	bar
	Fraction of NH_3	10, 20	%

Table S3. Kinetic parameters for reduction reactions by H_2, NH_3 and NH_3 decomposition.

	Reaction rate	Pre-exponential factor k_0 (m/s)	Apparent activation energy E_a (J/mol)	Equilibrium constant K_{eq}
Zone1 (<430°C)	$r_{1,i}$	0	0	-
Zone2 (430-570°C)	r_1	1.0×10^{-3} *	4.33×10^{4} *	Equation (S15)
	r_2	1.1×10^{2} *	1.12×10^{5} *	Equation (S15)
	r_3	6.3587×10^{-2} S2	43276 S2	$exp\left(-362.6\frac{T_{ref}}{T_{solids}}+10.334\right)$ S2
	r_4	0.14 *	4.65×10^{4} *	Equation (S16)
Zone3 (>570°C)	r_5	2.4×10^{-2} *	4.33×10^{4} *	Equation (S15)
	r_6	5.0×10^{4} *	1.58×10^{5} *	Equation (S15)
	r_7	0.1 *	9.93×10^{4} *	Equation (S15)

	r_8	6.3857×10^{-2} S2	43276 S2	$exp\left(-362.6\frac{T_{ref}}{T_{solids}}+10.334\right)$ S2
	r_9	1.129×10^{-3} S2	21570 [2]	$exp\left(-7916.6\frac{T_{ref}}{T_{solids}}+8.46\right)$ S2
	r_{10}	3.8337×10^{-2} S2	50129 [2]	$exp\left(-1586.9\frac{T_{ref}}{T_{solids}}+0.9317\right)$ S2
NH_3 decomposition	r_{11}***	3.35×10^{9} (mol/(s·kg)) S12	1.44×10^{5} S12	Equation (S16)
Nitridation	r_{12}	0.078 (1/s) S10	3.8×10^{4} S10	Equation (S17)
	r_{13}	0.109 (1/s) S10	4.3×10^{4} S10	Cube root of Equation (S18)
	r_{14}	88.730 (1/s) S10	1.0×10^{5} S10	Square root of Equation (S18)
	r_{15}	$k_{0,15}$** (1/s)	1.73×10^{5} S13	-

* Estimated from TGA experiments

** $k_{0,15} = -5.63129 \times 10^6 + 7090 \times T$ derived from Figure14a in Reference (S14).

*** Further parameters used in calculating r11 (Equation 34): α = 0.5; f_3 = 0.261; K_3 calculated from Reference (S12)

REFERENCES

(S1) Wakao, N.; Funazkri, T. Effect of fluid dispersion coefficients on particle-to-fluid mass transfer coefficients in packed beds, correlation of Sherwood numbers. *Chem. Eng. Sci.* **1978**, *33*, 1375-1384.

https://doi.org/10.1016/0009-2509(78)85120-3

(S2) Wagner, D. Étude expérimentale et modélisation de la réduction du minerai der fer par I’hydrogène. *PhD thesis*, Nancy-Université, France, 2008.

https://theses.hal.science/tel-00280689/

(S3) Fu, D.; Chen, Y.; Zhao, Y.; D'Alessio, J.; Ferron, K.J.; Zhou, C.Q. CFD modeling of multiphase reacting flow in blast furnace shaft with layered burden. *Appl. Therm. Eng.* **2014**, *66*, 298-308.

https://doi.org/10.1016/j.applthermaleng.2014.01.065

(S4) Seshadri; da Silva Pereira, R.O. Comparison of formulae for determining heat transfer coefficient of packed beds. *Trans. ISIJ* **1986**, *26*, 604-610.

https://doi.org/10.2355/isijinternational1966.26.604

(S5) Metolina, P.; da Silva, A.L.; Dixon, A.G.; Guardani, R. Multiscale modeling of non-catalytic gas-solid reactions applied to the hydrogen direct reduction of iron ore in moving-bed reactor. *Int. J. Hydrogen Energy* **2024**, *62*, 1214-1230.

https://doi.org/10.1016/j.ijhydene.2024.03.151

(S6) da Costa, A.R.; Wagner, D.; Patisson, F. Modeling a new, low CO_2 emissions, hydrogen steelmaking process. *J. Clean. Prod.* **2013**, *46*, 27-35.

https://doi.org/10.1016/j.jclepro.2012.07.045

(S7) https://webbook.nist.gov (accessed 20 February 2026).

(S8) Cheng, C.-H.; Nguyen, M.-T.; Leu, T.-S.; Chang, I.-L.; Liao, M.-L.; Panin, S.V.; Panin, A.V. Magnetic and mechanical properties of deformed iron nitride γ'-Fe_4N. *J. Appl. Math.* **2015**, 238730.

https://doi.org/10.1155/2015/238730

(S9) Chen, J.-S.; Yu, C.; Lu, H. Phase stability, magnetism, elastic properties and hardness of binary iron nitrides from first principles. *J. Alloys Compd.* **2015**, *625*, 224-230.

https://doi.org/10.1016/j.jallcom.2014.11.130

(S10) Arabczyk, W.; Zamłynny, J.; Moszyński, D. Kinetics of nanocrystalline iron nitriding. *Pol. J. Chem. Technol.* **2010**, *12*, 38-43.

https://doi.org/10.2478/v10026-010-0008-z

(S11) Satoh, S. Two iron nitrides. *Bull. Chem. Soc. Jpn.* **1932**, *7*, 315-333.

https://doi.org/10.1246/bcsj.7.315

(S12) Cholewa, T.; Steinbach, B.; Heim, C.; Nestler, F.; Nanba, T.; Güttel, R.; Salem, O. Reaction kinetics for ammonia synthesis using ruthenium and iron based catalysts under low temperature and pressure conditions. *Sustain. Energy Fuels* **2024**, *8*, 2245.

https://doi.org/10.1039/d4se00254g

(S13) Purcel, M.; Berendts, S.; Bonati, L.; Perego, S.; Müller, A.; Lerch, M.; Parrinello, M.; Muhler, M. Iron nitride formation and decomposition during ammonia decomposition over a wustite-based bulk iron catalyst. *ACS Catal.* **2024**, *14*, 13947-13957.

https://doi.org/10.1021/acscatal.4c04415

(S14) Widenmeyer, M.; Niewa, R.; Hansen, T.C.; Kohlmann, H. *In situ* Neutron Diffraction as a probe on formation and decomposition of nitrides and hydrides: a case study. *Z. Anorg. Allg. Chem.* **2013**, *639*, 285-295.

http://dx.doi.org/10.1002/zaac.201200299
